# Supplementary material for: Tissue-autonomous immune response regulates stress signaling during hypertrophy
Source: eLife. 2020 Dec 30;9:e64919. doi: 10.7554/eLife.64919 (PMC7880693; doi:10.7554/eLife.64919)
Supplement: Supplementary file 1. [file elife-64919-supp1.docx]

***Supplementary File 1***

***Figure 1***

**A-C**

☿ w^1118^,BxGal4;;UAS-GFP x ♂ w^1118^

☿ w^1118^,BxGal4;;UAS-GFP x ♂ w^1118^;;UAS-Ras^V12^

**D**

☿ w^1118^,BxGal4 x ♂ w^1118^

☿ w^1118^,BxGal4 x ♂ w^1118^;;UAS-Ras^V12^

**E-F**

☿ w^1118^,BxGal4;;Drs-GFP x ♂ w^1118^

☿ w^1118^,BxGal4;;Drs-GFP x ♂ w^1118^;;UAS-Ras^V12^

***Figure1-figure supplement 1***

**A**

☿ w^1118^,BxGal4,UAS-mCD8::RFP x ♂ w^1118^

☿ w^1118^,BxGal4,UAS-mCD8::RFP x ♂ w^1118^;;UAS-Ras^V12^

**B**

☿ w^1118^,BxGal4;;Drs-GFP x ♂ w^1118^

☿ w^1118^,BxGal4;;Drs-GFP x ♂ w^1118^;;UAS-Ras^V12^

**C**

☿ w^1118^,BxGal4;;UAS-GFP x ♂ w^1118^

☿ w^1118^,BxGal4;;UAS-GFP x ♂ w^1118^;;UAS-Ras^V12^

☿ w^1118^,BxGal4;;UAS-GFP x ♂ y,w^1118^;UAS-l(2)gl^RNAi^

☿ w^1118^,BxGal4;;UAS-GFP x ♂ w^1118^;UAS-l(2)gl^RNAi^;UAS-Ras^V12^

**D-F**

☿ w^1118^,BxGal4 x ♂ w^1118^

☿ w^1118^,BxGal4 x ♂ w^1118^;;UAS-Ras^V12^

☿ w^1118^,BxGal4 x ♂ y,w^1118^;UAS-l(2)gl^RNAi^

☿ w^1118^,BxGal4 x ♂ w^1118^;UAS-l(2)gl^RNAi^;UAS-Ras^V12^

**G-H**

☿ w^1118^,BxGal4;;Drs-GFP x ♂ w^1118^

☿ w^1118^,BxGal4;;Drs-GFP x ♂ w^1118^;;UAS-Ras^V12^

☿ w^1118^,BxGal4;;Drs-GFP x ♂ y,w^1118^;UAS-l(2)gl^RNAi^

☿ w^1118^,BxGal4;;Drs-GFP x ♂ w^1118^;UAS-l(2)gl^RNAi^;UAS-Ras^V12^

**I**

☿ w^1118^,BxGal4;;Drs-GFP x ♂ w^1118^;;UAS-Ras^V12^

***Figure 2***

**A**

☿ w^1118^,BxGal4;;Drs-GFP x ♂ w^1118^

☿ w^1118^,BxGal4;;Drs-GFP x ***♂*** w^1118^;;UAS-Ras^V12^

☿ w^1118^,BxGal4;;Drs-GFP x ♂ w^1118^;UAS-imd^RNAi^; UAS-Ras^V12^

☿ w^1118^,BxGal4;;Drs-GFP x ♂ w^1118^ ;UAS-Fadd^RNAi^; UAS-Ras^V12^

☿ w^1118^,BxGal4;;Drs-GFP x ♂ w^1118^;UAS-key^RNAi^; UAS-Ras^V12^

☿ w^1118^,BxGal4;;Drs-GFP x ♂ w^1118^;UAS-Rel^RNAi^; UAS-Ras^V12^

☿ w^1118^,BxGal4;;Drs-GFP x ♂ w^1118^;UAS-spz^RNAi^; UAS-Ras^V12^

☿ w^1118^,BxGal4;;Drs-GFP x ♂ w^1118^;UAS-Tl^RNAi^; UAS-Ras^V12^

☿ w^1118^,BxGal4;;Drs-GFP x ♂ w^1118^;UAS-Myd88^RNAi^; UAS-Ras^V12^

☿ w^1118^,BxGal4;;Drs-GFP x ♂ w^1118^;UAS-pll^RNAi^; UAS-Ras^V12^

☿ w^1118^,BxGal4;;Drs-GFP x ♂ w^1118^;UAS-Dif^RNAi^; UAS-Ras^V12^ (30578/GD)

☿ w^1118^,BxGal4;;Drs-GFP x ♂ w^1118^;UAS-Dif^RNAi^; UAS-Ras^V12^ (30579/GD)

☿ w^1118^,BxGal4;;Drs-GFP x ♂ w^1118^;UAS-dl^RNAi^; UAS-Ras^V12^

**B**

☿ w^1118^,BxGal4;;Drs-GFP x ♂ w^1118^

☿ w^1118^,BxGal4;;Drs-GFP x ***♂*** w^1118^;;UAS-Ras^V12^

☿ w^1118^,BxGal4;;Drs-GFP x ***♂*** y^1^,sc*,v^1^,sev^21^;UAS-dl^RNAi^

☿ w^1118^,BxGal4;;Drs-GFP x ***♂*** w^1118^;UAS-dl^RNAi^;UAS-Ras^V12^

**C**

☿ w^1118^,BxGal4 x ♂ w^1118^

☿ w^1118^,BxGal4 x ***♂*** w^1118^;;UAS-Ras^V12^

☿ w^1118^,BxGal4 x ♂ w^1118^;dl^15^/CyO,GFP;UAS-Ras^V12^

☿ w^1118^,BxGal4;dl^15^/CyO,GFP x ♂ w^1118^;dl^15^/CyO,GFP;UAS-Ras^V12^

***Figure 2-figure supplement 1***

**A-A’’**

☿ w^1118^,BxGal4;;Drs-GFP x ♂ w^1118^;;UAS-Ras^V12^

***Figure 2-figure supplement 2***

**A**

☿ w^1118^,BxGal4;;Drs-GFP x ♂ w^1118^

☿ w^1118^,BxGal4;;Drs-GFP x ♂ w^1118^;;UAS-Ras^V12^

☿ w^1118^,BxGal4;;Drs-GFP x ♂ w^1118^;UAS-cad^RNAi^;UAS-Ras^V12^

☿ w^1118^,BxGal4;;Drs-GFP x ♂ w^1118^;UAS-dfr^RNAi^;UAS-Ras^V12^

**B**

☿ w^1118^,BxGal4;;Drs-GFP x ***♂*** w^1118^;;UAS-Ras^V12^

**C**

☿ w^1118^,BxGal4 x ♂ w^1118^

☿ w^1118^,BxGal4 x ***♂*** w^1118^;;UAS-Ras^V12^

☿ w^1118^,BxGal4 x ***♂*** y^1^,sc*,v^1^,sev^21^;UAS-dl^RNAi^

☿ w^1118^,BxGal4 x ***♂*** w^1118^;UAS-dl^RNAi^;UAS-Ras^V12^

**D**

☿ w^1118^,BxGal4 x ♂ w^1118^

☿ w^1118^,BxGal4 x ♂ w^1118^;dl^15^/CyO,GFP

☿ w^1118^,BxGal4 x ***♂*** w^1118^;;UAS-Ras^V12^

☿ w^1118^,BxGal4 x ♂ w^1118^;dl^15^/CyO,GFP;UAS-Ras^V12^

☿ w^1118^,BxGal4;dl^15^/CyO,GFP x ♂ w^1118^ ;dl^15^/CyO,GFP

☿ w^1118^,BxGal4;dl^15^/CyO,GFP x ♂ w^1118^;dl^15^/CyO,GFP;UAS-Ras^V12^

**E**

☿ w^1118^,BxGal4 x ♂ w^1118^

☿ w^1118^,BxGal4 x ♂ w^1118^;Myd88^KG03447^/CyO,GFP

☿ w^1118^,BxGal4 x ***♂*** w^1118^;;UAS-Ras^V12^

☿ w^1118^,BxGal4 x ♂ w^1118^;Myd88^KG03447^/CyO,GFP;UAS-Ras^V12^

☿ w^1118^,BxGal4;Myd88^KG03447^/CyO,GFP x ♂ w^1118^ ;Myd88^KG03447^/CyO,GFP

☿ w^1118^,BxGal4;Myd88^KG03447^/CyO,GFP x ♂ w^1118^;Myd88^KG03447^/CyO,GFP;UAS-Ras^V12^

***Figure 2-figure supplement 3***

**A**

☿ w^1118^,BxGal4;;Drs-GFP x ♂ w^1118^;;UAS-Ras^V12^

☿ w^1118^,BxGal4;;Drs-GFP x ♂ w^1118^;UAS-dl^RNAi^;UAS-Ras^V12^

**B**

☿ w^1118^,BxGal4 x ♂ w^1118^

☿ w^1118^,BxGal4 x ♂ w^1118^;;UAS-Ras^V12^

☿ w^1118^,BxGal4;;Drs-GFP x ♂ w^1118^;UAS-l(2)gl^RNAi^;UAS-Ras^V12^

**C**

☿ w^1118^,BxGal4;;Drs-GFP x ♂ w^1118^

☿ w^1118^,BxGal4;;Drs-GFP x ♂ w^1118^;;UAS-Ras^V12^

☿ w^1118^,BxGal4;;Drs-GFP x ♂ w^1118^;UAS-Stat92E ^RNAi^;UAS-Ras^V12^

☿ w^1118^,BxGal4;;Drs-GFP x ♂ w^1118^;UAS-foxo ^RNAi^;UAS-Ras^V12^

☿ w^1118^,BxGal4;;Drs-GFP x ♂ w^1118^;UAS-grh^RNAi^;UAS-Ras^V12^

☿ w^1118^,BxGal4;;Drs-GFP x ♂ w^1118^;UAS-Mef2^RNAi^;UAS-Ras^V12^

☿ w^1118^,BxGal4;;Drs-GFP x ♂ w^1118^;UAS-Nrf2^RNAi^;UAS-Ras^V12^ (101235/KK)

☿ w^1118^,BxGal4;;Drs-GFP x ♂ w^1118^;UAS-Nrf2^RNAi^;UAS-Ras^V12^ (108127/GD)

☿ w^1118^,BxGal4;;Drs-GFP x ♂ w^1118^;UAS-Sox14^RNAi^;UAS-Ras^V12^

***Figure 3***

**A-E**

☿ w^1118^,BxGal4 x ♂ w^1118^

☿ w^1118^,BxGal4 x ♂ w^1118^;;UAS-Ras^V12^

☿ w^1118^,BxGal4;;Drs-GFP x ♂ w^1118^;UAS-l(2)gl^RNAi^;UAS-Ras^V12^

**F**

☿ w^1118^,BxGal4;TREGFP1b/CyO,GFP x ♂ w^1118^

☿ w^1118^,BxGal4;TREGFP1b/CyO,GFP x ***♂*** w^1118^;;UAS-Ras^V12^

***Figure 3-figure supplement 1***

**A-C**

☿ w^1118^,BxGal4 x ♂ w^1118^

☿ w^1118^,BxGal4 x ♂ w^1118^;;UAS-Ras^V12^

☿ w^1118^,BxGal4;;Drs-GFP x ♂ w^1118^;UAS-l(2)gl^RNAi^;UAS-Ras^V12^

**D**

☿ w^1118^,BxGal4;;Drs-GFP x ♂ w^1118^

☿ w^1118^,BxGal4;;Drs-GFP x ♂ w^1118^;;UAS-Ras^V12^

**E**

☿ w^1118^,BxGal4 x ♂ w^1118^

☿ w^1118^,BxGal4 x ♂ w^1118^;;UAS-Ras^V12^

☿ w^1118^,BxGal4 x ***♂*** y^1^,sc*,v^1^,sev^21^;UAS-dl^RNAi^

☿ w^1118^,BxGal4 x ***♂*** w^1118^;UAS-dl^RNAi^;UAS-Ras^V12^

☿ w^1118^,BxGal4 x ♂ w^1118^;UAS-CecA1

☿ w^1118^,BxGal4 x ♂ w^1118^;UAS-CecA1;UAS-Ras^V12^

**F**

☿ w^1118^,BxGal4;10xStat92E-GFP x ♂ w^1118^

☿ w^1118^,BxGal4;10xStat92E-GFP x ♂ w^1118^;;UAS-Ras^V12^

☿ w^1118^,BxGal4;10xStat92E-GFP x ♂ w^1118^;UAS-l(2)gl^RNAi^;UAS-Ras^V12^

***Figure 3-figure supplement 2***

**A**

☿ w^1118^,BxGal4 x ♂ w^1118^

☿ w^1118^,BxGal4 x ♂ w^1118^;;UAS-Ras^V12^

☿ w^1118^,BxGal4 x ♂ w^1118^;UAS-l(2)gl^RNAi^;UAS-Ras^V12^

**B**

☿ w^1118^,BxGal4;TREGFP1b/CyO,GFP x ♂ w^1118^

☿ w^1118^,BxGal4;TREGFP1b/CyO,GFP x ***♂*** w^1118^;;UAS-Ras^V12^

☿ w^1118^,BxGal4;TREGFP1b/CyO,GFP x ♂ y,w^1118^;UAS-l(2)gl^RNAi^

☿ w^1118^,BxGal4;TREGFP1b/CyO,GFP x ♂ w^1118^;UAS-l(2)gl^RNAi^;UAS-Ras^V12^

***Figure 4***

**A**

☿ w^1118^,BxGal4 x ♂ w^1118^

☿ w^1118^,BxGal4 x ♂ w^1118^;;UAS-Ras^V12^

☿ w^1118^,BxGal4 x ♂ w^1118^;;UAS-Drs

☿ w^1118^,BxGal4 x ♂ w^1118^;;UAS-Drs,UAS-Ras^V12^

☿ w^1118^,BxGal4 x ♂ w^1118^,UAS-jnk^DN^

☿ w^1118^,BxGal4 x ♂ w^1118^,UAS-jnk^DN^;; UAS-Ras^V12^

**B-D**

☿ w^1118^,BxGal4;vkg^G00454^/CyO,GFP x ♂ w^1118^

☿ w^1118^,BxGal4;vkg^G00454^/CyO,GFP x ♂ w^1118^;;UAS-Ras^V12^

☿ w^1118^,BxGal4;vkg^G00454^/CyO,GFP x ♂ w^1118^;;UAS-Drs

☿ w^1118^,BxGal4;vkg^G00454^/CyO,GFP x ♂ w^1118^;;UAS-Drs,UAS-Ras^V12^

☿ w^1118^,BxGal4;vkg^G00454^/CyO,GFP x ♂ w^1118^,UAS-jnk^DN^

☿ w^1118^,BxGal4;vkg^G00454^/CyO,GFP x ♂ w^1118^,UAS-jnk^DN^;; UAS-Ras^V12^

**E**

☿ w^1118^,BxGal4 x ♂ w^1118^

☿ w^1118^,BxGal4 x ♂ w^1118^;;UAS-Ras^V12^

**F-G**

☿ w^1118^,BxGal4 x ♂ w^1118^

☿ w^1118^,BxGal4 x ♂ w^1118^;;UAS-Ras^V12^

☿ w^1118^,BxGal4;Mmp2^k00604^/CyO,GFP x ♂ w^1118^

☿ w^1118^,BxGal4;Mmp2^k00604^/CyO,GFP x ♂ w^1118^;;UAS-Ras^V12^

☿ w^1118^,BxGal4;Mmp2^k00604^/CyO,GFP x ♂ w^1118^;Mmp2^k00604^/CyO,GFP;UAS-Ras^V12^

***Figure 4-figure supplement 1***

**A**

☿ w^1118^,BxGal4 x ♂ w^1118^

☿ w^1118^,BxGal4,UAS-mCD8::RFP x ♂ w^1118^

☿ w^1118^,BxGal4 x ♂ w^1118^;;UAS-Ras^V12^

☿ w^1118^,BxGal4,UAS-mCD8::RFP x ♂ w^1118^;;UAS-Ras^V12^

☿ w^1118^,BxGal4,UAS-mCD8::RFP x ♂ w^1118^;;UAS-Drs

☿ w^1118^,BxGal4 x ♂ w^1118^;;UAS-Drs,UAS-Ras^V12^

**B**

☿ w^1118^,BxGal4;vkg^G00454^/CyO,GFP x ♂ w^1118^

☿ w^1118^,BxGal4;vkg^G00454^/CyO,GFP x ♂ w*;UAS-Mmp1^APM3099^/CyO,Tb

☿ w^1118^,BxGal4;vkg^G00454^/CyO,GFP x ♂ w*;UAS-Mmp1^APM1037^/TM3

☿ w^1118^,BxGal4;vkg^G00454^/CyO,GFP x ♂ w*;UAS-Mmp2^#4^/CyO

**C**

☿ w^1118^,BxGal4 x ♂ w^1118^

☿ w^1118^,BxGal4 x ♂ w^1118^;;UAS-Ras^V12^

☿ w^1118^,BxGal4;Mmp2^k00604^/CyO,GFP x ♂ w^1118^

☿ w^1118^,BxGal4;Mmp2^k00604^/CyO,GFP x ♂ w^1118^;;UAS-Ras^V12^

☿ w^1118^,BxGal4;Mmp2^k00604^/CyO,GFP x ♂ w^1118^;Mmp2^k00604^/CyO,GFP;UAS-Ras^V12^

**D**

☿ w^1118^,BxGal4 x ♂ w^1118^

☿ w^1118^,BxGal4 x ♂ w^1118^;;UAS-Ras^V12^

☿ w^1118^,BxGal4 x ♂ w^1118^;;UAS-Drs

☿ w^1118^,BxGal4 x ♂ w^1118^;;UAS-Drs,UAS-Ras^V12^

☿ w^1118^,BxGal4 x ♂ w^1118^,UAS-jnk^DN^

☿ w^1118^,BxGal4 x ♂ w^1118^,UAS-jnk^DN^;; UAS-Ras^V12^

***Figure 5***

**B**

☿ w^1118^,BxGal4 x ♂ w^1118^

☿ w^1118^,BxGal4 x ♂ w^1118^;;UAS-Ras^V12^

☿ w^1118^,BxGal4 x ♂ w^1118^;;UAS-Drs

☿ w^1118^,BxGal4 x ♂ w^1118^;;UAS-Drs,UAS-Ras^V12^

**C-F**

☿ w^1118^,BxGal4;;TREGFP1b ♂ w^1118^

☿ w^1118^,BxGal4;;TREGFP1b x ♂ w^1118^;;UAS-Ras^V12^

☿ w^1118^,BxGal4;;TREGFP1b x ♂ w^1118^;;UAS-Drs

☿ w^1118^,BxGal4;;TREGFP1b x ♂ w^1118^;;UAS-Drs,UAS-Ras^V12^

***Figure 5-figure supplement 1***

**A**

☿ w^1118^,BxGal4;;Drs-GFP ♂ w^1118^

☿ w^1118^,BxGal4;;Drs-GFP x ♂ w^1118^;;UAS-Ras^V12^

☿ w^1118^,BxGal4;;Drs-GFP x ♂ w^1118^,UAS-jnk^DN^

☿ w^1118^,BxGal4;;Drs-GFP x ♂ w^1118^,UAS-jnk^DN^;; UAS-Ras^V12^

**B**

☿ w^1118^,BxGal4 x ♂ w^1118^

☿ w^1118^,BxGal4 x ♂ w^1118^;;UAS-Ras^V12^

☿ w^1118^,BxGal4 x ♂ w^1118^,UAS-jnk^DN^

☿ w^1118^,BxGal4 x ♂ w^1118^,UAS-jnk^DN^;; UAS-Ras^V12^

***Figure 5- figure supplement 2***

**A**

☿ w^1118^,BxGal4 x ♂ w^1118^;;UAS-Ras^V12^

☿ w^1118^,BxGal4 x ♂ w^1118^;UAS-Drs^RNAi^;UAS-Ras^V12^

**B**

☿ w^1118^,BxGal4 x ♂ w^1118^

☿ w^1118^,BxGal4 x ♂ w^1118^;;UAS-Ras^V12^

☿ w^1118^,BxGal4 x ♂ w^1118^;UAS-Drs^RNAi^

☿ w^1118^,BxGal4 x ♂ w^1118^;UAS-Drs^RNAi^;UAS-Ras^V12^

**C-D**

☿ w^1118^,BxGal4 x ♂ w^1118^

☿ w^1118^,BxGal4 x ♂ w^1118^;;UAS-Ras^V12^

☿ w^1118^,BxGal4 x ♂ w^1118^;;UAS-Drs

☿ w^1118^,BxGal4 x ♂ w^1118^;;UAS-Drs,UAS-Ras^V12^

☿ w^1118^,BxGal4 x ♂ w^1118^;UAS-Drs^RNAi^;UAS-Ras^V12^

**E-H**

☿ w^1118^,BxGal4;TREGFP1b/CyO,GFP x ♂ w^1118^

☿ w^1118^,BxGal4;TREGFP1b/CyO,GFP x ♂ w^1118^;;UAS-Ras^V12^

☿ w^1118^,BxGal4;TREGFP1b/CyO,GFP x ♂ w^1118^;;UAS-Drs

☿ w^1118^,BxGal4;TREGFP1b/CyO,GFP x ♂ w^1118^;;UAS-Drs,UAS-Ras^V12^

☿ w^1118^,BxGal4;TREGFP1b/CyO,GFP x ♂ w^1118^;UAS-Drs^RNAi^

☿ w^1118^,BxGal4;TREGFP1b/CyO,GFP x ♂ w^1118^;UAS-Drs^RNAi^;UAS-Ras^V12^

***Figure 5-figure supplement 3***

**A-C**

☿ w^1118^,BxGal4 x ♂ w^1118^

☿ w^1118^,BxGal4,UAS-mCD8::RFP x ♂ w^1118^

☿ w^1118^,BxGal4 x ♂ w^1118^;;UAS-Ras^V12^

☿ w^1118^,BxGal4,UAS-mCD8::RFP x ♂ w^1118^;;UAS-Ras^V12^

☿ w^1118^,BxGal4,UAS-mCD8::RFP x ♂ w^1118^;;UAS-Drs

☿ w^1118^,BxGal4 x ♂ w^1118^;;UAS-Drs,UAS-Ras^V12^

***Figure 6***

**A**

☿ w^1118^,BxGal4 x ♂ w^1118^

☿ w^1118^,BxGal4 x ♂ w^1118^;;UAS-Ras^V12^

☿ w^1118^,BxGal4 x ♂ w^1118^;;UAS-Drs

☿ w^1118^,BxGal4 x ♂ w^1118^;;UAS-Drs,UAS-Ras^V12^

☿ w^1118^,BxGal4 x ♂ w^1118^;UAS-Drs^RNAi^;UAS-Ras^V12^

**C-D**

☿ w^1118^,BxGal4 x ♂ w^1118^

☿ w^1118^,BxGal4 x ♂ w^1118^;;UAS-Ras^V12^

☿ w^1118^,BxGal4 x ♂ w^1118^,UAS-jnk^DN^

☿ w^1118^,BxGal4 x ♂ w^1118^,UAS-jnk^DN^;; UAS-Ras^V12^

☿ w^1118^,BxGal4 x ♂ w^1118^;UAS-p35

☿ w^1118^,BxGal4 x ♂ w^1118^;UAS-p35;UAS-Ras^V12^

☿ w^1118^,BxGal4 x ♂ w^1118^;;UAS-Drs

☿ w^1118^,BxGal4 x ♂ w^1118^;;UAS-Drs,UAS-Ras^V12^

**E-F**

☿ w^1118^,BxGal4;TREGFP1b/CyO,GFP x ♂ x w^1118^

☿ w^1118^,BxGal4;TREGFP1b/CyO,GFP x ♂ w^1118^;;UAS-Ras^V12^

☿ w^1118^,BxGal4;TREGFP1b/CyO,GFP x ♂ w^1118^;;UAS-Drs

☿ w^1118^,BxGal4;TREGFP1b/CyO,GFP x ♂ w^1118^;;UAS-Drs,UAS-Ras^V12^

***Figure 6-figure supplement 1***

**A-B**

☿ w^1118^,BxGal4;TREGFP1b/CyO,GFP x ♂ w^1118^

☿ w^1118^,BxGal4;TREGFP1b/CyO,GFP x ♂ w^1118^;;UAS-Ras^V12^

☿ w^1118^,BxGal4;TREGFP1b/CyO,GFP x ♂ w^1118^;;UAS-Drs

☿ w^1118^,BxGal4;TREGFP1b/CyO,GFP x ♂ w^1118^;;UAS-Drs,UAS-Ras^V12^

☿ w^1118^,BxGal4;TREGFP1b/CyO,GFP x ♂ w^1118^;;UAS-Drs^RNAi^

☿ w^1118^,BxGal4;TREGFP1b/CyO,GFP x ♂ w^1118^;UAS-Drs^RNAi^;UAS-Ras^V12^

**C-D**

☿ w^1118^,BxGal4 x ♂ w^1118^

☿ w^1118^,BxGal4,UAS-mCD8::RFP x ♂ w^1118^

☿ w^1118^,BxGal4 x ♂ w^1118^;;UAS-Ras^V12^

☿ w^1118^,BxGal4,UAS-mCD8::RFP x ♂ w^1118^;;UAS-Ras^V12^

☿ w^1118^,BxGal4,UAS-mCD8::RFP x ♂ w^1118^;;UAS-Drs

☿ w^1118^,BxGal4 x ♂ w^1118^;;UAS-Drs,UAS-Ras^V12^

**E-F**

☿ w^1118^,BxGal4 x ♂ w^1118^

☿ w^1118^,BxGal4 x ♂ w^1118^;;UAS-Ras^V12^

☿ w^1118^,BxGal4;;UAS-hid^RNAi^/TM6B x ♂ w^1118^

☿ w^1118^,BxGal4;;UAS-hid^RNAi^/TM6B x ♂ w^1118^;;UAS-Ras^V12^

***Figure 7***

**B-C,E**

☿ w^1118^,BxGal4;tubGal80^ts^ x ♂ w^1118^

☿ w^1118^,BxGal4;tubGal80^ts^ x ♂ w^*^; UAS-hid

☿ w^1118^,BxGal4;tubGal80^ts^ x ♂ w^1118^;;UAS-Drs

☿ w^1118^,BxGal4;tubGal80^ts^ x ♂ w^1118^;UAS-hid;UAS-Drs

**D,F**

☿ w^1118^,BxGal4 x ♂ w^1118^

☿ w^1118^,BxGal4 x ♂ w^1118^;;UAS-Ras^V12^

☿ w^1118^,BxGal4 x ♂ w^1118^;UAS-Dronc^RNAi^ (23035/GD)

☿ w^1118^,BxGal4 x ♂ w^1118^;UAS-Dronc^RNAi^; UAS-Ras^V12^ (23035/GD)

***Figure 7-figure supplement 1***

**A-B**

☿ w^1118^,BxGal4 x ♂ w^1118^

☿ w^1118^,BxGal4 x ♂ w^1118^;;UAS-Ras^V12^

☿ w^1118^,BxGal4 x ♂ w^1118^;UAS-Dronc^RNAi^ (23035/GD)

☿ w^1118^,BxGal4 x ♂ w^1118^;UAS-Dronc^RNAi^;UAS-Ras^V12^ (23035/GD)

☿ w^1118^,BxGal4 x ♂ w^1118^;UAS-Dronc^RNAi^ (100424/KK)

☿ w^1118^,BxGal4 x ♂ w^1118^,UAS-Dronc^RNAi^;UAS-Ras^V12^ (100424/KK)
