# Supplementary material for: Tissue-autonomous immune response regulates stress signaling during hypertrophy
Source: eLife. 2020 Dec 30;9:e64919. doi: 10.7554/eLife.64919 (PMC7880693; doi:10.7554/eLife.64919)
Supplement: Supplementary file 2. [file elife-64919-supp2.docx]

***Supplementary File 2.***

| ***Gene*** | ***qPCR forward primer*** | ***qPCR reverse primer*** |
| --- | --- | --- |
| Drosomycin | 5’-gaggagggacgctccagt-3’ | 5’-ttagcatccttcgcaccag-3’ |
| AttD | 5'-gtttatggagcggtcaacg-3' | 5'-tctggaagagattggcttgg-3' |
| TIMP | 5’-aacagagcgtcatggcttca-3’ | 5’-tcacaccaaaacaggtggca-3’ |
| Upd1 | 5’-cgggtgatcgcttcaatc-3’ | 5’-ctgcggtactcccgaaag-3’ |
| Upd2 | 5’-aagttcctgccgaacatgac-3’ | 5’-atccttgcggaacttgtactg-3’ |
| Upd3 | 5’-actgggagaacacctgcaat-3’ | 5’-gcccgtttggttctgtagat-3’ |
| Hid | 5’-tctacgagtgggtcaggatgt-3’ | 5’-gcggatactggaagatttgc-3’ |
| Reaper | 5’-gatcaggcgactctgttgc-3’ | 5’-actgtgactcccgcaagc-3’ |
| Grim | 5’-atcgatgaccatgtcggagt-3’ | 5’-cgcagagcgtagcagaagat-3’ |
| Mmp1 | 5’-gtttccaccaccacacagg-3’ | 5’-gcagaggcgggtagatagc-3’ |
| Mmp2 | 5’-tttcgatgcggacgagac-3’ | 5’-gccacgttcagaaaattggt-3’ |
| Puc | 5’-cgtcatcatcaacggcaat-3’ | 5’-aggcggggtgtgtttctat-3’ |
| Rpl32 | 5’-cggatcgatatgcta-3’ | 5’-cgacgcactctgttg-3’ |
