# Supplementary material for: Tissue-autonomous immune response regulates stress signaling during hypertrophy
Source: eLife. 2020 Dec 30;9:e64919. doi: 10.7554/eLife.64919 (PMC7880693; doi:10.7554/eLife.64919)
Supplement: Supplementary file 3. [file elife-64919-supp3.docx]

***Supplementary File 3.***

| ***Transgenic line*** | ***Source*** | ***Identifier*** | ***Reference*** |
| --- | --- | --- | --- |
| UAS-dl^RNAi^ | Bloomington | 36650 |  |
| UAS-l(2)gl^RNAi^ | VDRC | 109604/KK | (107) |
| UAS-imd^RNAi^ | VDRC | 101834/KK | (108) |
| UAS-Fadd^RNAi^ | VDRC | 100333/KK |  |
| UAS-key^RNAi^ | VDRC | 100257/KK |  |
| UAS-Rel^RNAi^ | VDRC | 108469/KK | (109) |
| UAS-spz^RNAi^ | VDRC | 105017/KK | (110) |
| UAS-Tl^RNAi^ | VDRC | 100078/KK | (111) |
| UAS-cad^RNAi^ | VDRC | 49562/KK |  |
| UAS-Stat92E^RNAi^ | VDRC | 106980/KK | (112) |
| UAS-Dronc^RNAi^ | VDRC | 100424/KK | (113) |
| UAS-Dronc^RNAi^ | VDRC | 23035/GD | (114) |
| UAS-Myd88^RNAi^ | VDRC | 25402/GD | (115) |
| UAS-pll^RNAi^ | VDRC | 2889/GD |  |
| UAS-Dif^RNAi^ | VDRC | 30578/GD |  |
| UAS-Dif^RNAi^ | VDRC | 30579/GD |  |
| UAS-Drs^RNAi^ | VDRC | 2703/GD |  |
| UAS-dfr^RNAi^ | S. Certel |  |  |
| UAS-hid^RNAi^ | VDRC | 8269/GD | (116) |
| UAS-foxo^RNAi^ | VDRC | 107786/KK | (117) |
| UAS-grh^RNAi^ | VDRC | 33680/GD |  |
| UAS-Mef2^RNAi^ | Bloomington | 38247 | (118) |
| UAS-Nrf2^RNAi^ | VDRC | 101235/KK | (119) |
| UAS-Nrf2^RNAi^ | VDRC | 108127/KK | (119) |
| UAS-Sox14^RNAi^ | VDRC | 107146/KK | (120) |
