# Supplementary material for: Tissue-autonomous immune response regulates stress signaling during hypertrophy
Source: eLife. 2020 Dec 30;9:e64919. doi: 10.7554/eLife.64919 (PMC7880693; doi:10.7554/eLife.64919)
Supplement: Transparent reporting form [file elife-64919-transrepform.docx]

***eLife’s* transparent reporting form**

We encourage authors to provide detailed information *within their submission* to facilitate the interpretation and replication of experiments. Authors can upload supporting documentation to indicate the use of appropriate reporting guidelines for health-related research (see [EQUATOR Network](http://www.equator-network.org/%20)), life science research (see the [BioSharing Information Resource](https://biosharing.org/)), or the [ARRIVE guidelines](http://www.plosbiology.org/article/info:doi/10.1371/journal.pbio.1000412) for reporting work involving animal research. Where applicable, authors should refer to any relevant reporting standards documents in this form.

If you have any questions, please consult our Journal Policies and/or contact us: [editorial@elifesciences.org](mailto:editorial@elifesciences.org).

**Sample-size estimation**

- You should state whether an appropriate sample size was computed when the study was being designed
- You should state the statistical method of sample size computation and any required assumptions
- If no explicit power analysis was used, you should describe how you decided what sample (replicate) size (number) to use

Please outline where this information can be found within the submission (e.g., sections or figure legends), or explain why this information doesn’t apply to your submission:

Corresponding information and clarifications can be found in the ‘Materials and methods’ section, i.e., line 609-611 for RNAseq data acquisition, line 683-687 and 698-699 for quantifications of fluorescence signals from Immunohistochemistry and line 757-759 for in-situ hybridizations.

**Replicates**

- You should report how often each experiment was performed
- You should include a definition of biological versus technical replication
- The data obtained should be provided and sufficient information should be provided to indicate the number of independent biological and/or technical replicates
- If you encountered any outliers, you should describe how these were handled
- Criteria for exclusion/inclusion of data should be clearly stated
- High-throughput sequence data should be uploaded before submission, with a private link for reviewers provided (these are available from both GEO and ArrayExpress)

Please outline where this information can be found within the submission (e.g., sections or figure legends), or explain why this information doesn’t apply to your submission:

For a definition of ‘replicate’ see ‘Staging’-paragraph in ‘Materials and methods’-section, especially line 572-575. Outliers were not excluded, see line 687. All replicates for quantitative data are either shown as individual points throughout the graphs (e.g., Fig1C.,F. for nuclei volume and hemocyte attachment) or their amount is delineated underneath the graph for qPCR data and the DrsGFP screen (i.e., Fig3E., Fig2A.). The RNAseq data can be accessed via NCBI GEO under the record GSE138936 (reviewer token: abyziywirncphir).

**Statistical reporting**

- Statistical analysis methods should be described and justified
- Raw data should be presented in figures whenever informative to do so (typically when N per group is less than 10)
- For each experiment, you should identify the statistical tests used, exact values of N, definitions of center, methods of multiple test correction, and dispersion and precision measures (e.g., mean, median, SD, SEM, confidence intervals; and, for the major substantive results, a measure of effect size (e.g., Pearson's r, Cohen's d)
- Report exact p-values wherever possible alongside the summary statistics and 95% confidence intervals. These should be reported for all key questions and not only when the p-value is less than 0.05.

Please outline where this information can be found within the submission (e.g., sections or figure legends), or explain why this information doesn’t apply to your submission:

Information regarding descriptive statistics is given in every figure legend for panels representing quantitative data (e.g., Fig1C.,F.) and for statistical testing in the ‘Materials and methods’-section, i.e., line 589-590 for DrsGFP-assay, line 617-618 and 627-629 for RNAseq, line 629-631 and 641-643 for motif enrichment, line 677-683 and 698-699 for fluorescence signals, line 707-709 for Drs-dl-correlation and line 722-723 for driver expression strength.

(For large datasets, or papers with a very large number of statistical tests, you may upload a single table file with tests, Ns, etc., with reference to sections in the manuscript.)

**Group allocation**

- Indicate how samples were allocated into experimental groups (in the case of clinical studies, please specify allocation to treatment method); if randomization was used, please also state if restricted randomization was applied
- Indicate if masking was used during group allocation, data collection and/or data analysis

Please outline where this information can be found within the submission (e.g., sections or figure legends), or explain why this information doesn’t apply to your submission:

Neither randomization, nor masking were applied throughout this study. Groups were determined by genotype and delineated in detail in Supplemental File 2.

**Additional data files (“source data”)**

- We encourage you to upload relevant additional data files, such as numerical data that are represented as a graph in a figure, or as a summary table
- Where provided, these should be in the most useful format, and they can be uploaded as “Source data” files linked to a main figure or table
- Include model definition files including the full list of parameters used
- Include code used for data analysis (e.g., R, MatLab)
- Avoid stating that data files are “available upon request”

Please indicate the figures or tables for which source data files have been provided:

The entire workflow detailing the analysis of the RNAseq data and the detection of enriched motifs and Mef2-motif distribution are deposited on GitHub (<https://github.com/robertkrautz/sg_analysis>). The ImageJ-macro devised to quantify the fluorescence signal along the longitudinal axis of glands with the name “scanner.ijm” is available in the same repository.
